# Plasma cell free DNA methylation markers for hepatocellular carcinoma surveillance in patients with cirrhosis: a case control study

**DOI:** 10.1186/s12876-021-01714-8

**Published:** 2021-03-25

**Authors:** Jörn Lewin, Denise Kottwitz, Johanna Aoyama, Theo deVos, Jorge Garces, Oliver Hasinger, Stefanie Kasielke, Florian Knaust, Preeti Rathi, Sebastian Rausch, Gunter Weiss, Alexander Zipprich, Edward Mena, Tse-Ling Fong

**Affiliations:** 1 Epigenomics AG, Geneststr. 5, 10829 Berlin, Germany; 2 Epigenomics Inc., 11055 Flintkote Ave, Suite A, San Diego, CA 92121 USA; 3 Universitätsklinik und Poliklinik für Innere Medizin I, UKH Martin-Luther-Universität Halle-Wittenberg, Ernst-Grube-Str. 40, 06120 Halle (Saale), Germany; 4 California Liver Research Institute, 301 S. Fair Oaks Avenue, Suite 409, Pasadena, CA 91105 USA; 5 Keck School of Medicine, USC, 1510 San Pablo Street, 2/F, Los Angeles, CA 90033 USA

**Keywords:** Carcinoma, Hepatocellular, Liver cirrhosis, Early detection of cancer, Biomarker, Cell-free nucleic acids, DNA methylation

## Abstract

**Background:**

Hepatocellular carcinoma (HCC) is the leading cause of death in patients with cirrhosis, primarily due to failed early detection. HCC screening is recommended among individuals with cirrhosis using biannual abdominal ultrasound, for earlier tumor detection, administration of curative treatment, and improved survival. Surveillance by imaging with or without biomarkers such as alpha-fetoprotein (AFP) remains suboptimal for early stage HCC detection. Here we report on the development and assessment of methylation biomarkers from liquid biopsies for HCC surveillance in cirrhotic patients.

**Methods:**

DNA methylation markers including the HCCBloodTest (Epigenomics AG) and a DNA-methylation panel established by next generation sequencing (NGS) were assessed using a training/testing design. The NGS panel algorithm was established in a training study (41 HCC patients; 46 cirrhotic non-HCC controls). For testing, plasma samples were obtained from cirrhotic patients (Child class A or B) with (60) or without (103) early stage HCC (BCLC stage 0, A, B). The assays were then tested using blinded sample sets and analyzed by preset algorithms.

**Results:**

The HCCBloodTest and the NGS panel exhibited 76.7% and 57% sensitivities at 64.1% and 97% specificity, respectively. In a post-hoc analysis, a combination of the NGS panel with AFP (20 ng/mL) achieved 68% sensitivity at 97% specificity (AUC = 0.9).

**Conclusions:**

Methylation biomarkers in cell free plasma DNA provide a new alternative for HCC surveillance. Multiomic panels comprising DNA methylation markers with other biological markers, such as AFP, provide an option to further increase the overall clinical performance of surveillance via minimally invasive blood samples.

*Trial Registration*: Test set study—ClinicalTrials.gov (NCT03804593) January 11, 2019, retrospectively registered.

**Supplementary Information:**

The online version contains supplementary material available at 10.1186/s12876-021-01714-8.

## Background

Hepatocellular carcinoma (HCC) is the 5th most common cancer in men, and 9th in women worldwide with an incidence of more than 840,000 cases and 780,000 deaths annually (2018) [1]. In the US an estimated 42,000 new cases and 30,000 deaths due to HCC will occur in 2020 [2]. Most HCC cases occur in patients with cirrhosis, a condition affecting an estimated 4.5 million Americans [3]. The diagnosis of HCC at an early stage offers the chance of curative treatment whereas regional and metastatic disease is associated with a 5-year survival rates of 11% and 3% respectively [2]. To identify HCC at an early treatable stage, the American Association for the Study of Liver Diseases (AASLD) recommends surveillance of adults with cirrhosis by ultrasound with or without alpha-fetoprotein (AFP) every 6 months [4].

The AASLD guideline recommendation regarding AFP was based in part on an improvement in sensitivity of HCC detection when AFP was used in conjunction with ultrasound (US). Though the data is limited, performance of combined US + AFP is still not optimal for early detection, with a reported sensitivity of 63% at a pooled specificity of 84% [5, 6]. Given the size of the at risk cirrhotic population, the effort required for guideline recommended surveillance, the current suboptimal performance of recommended surveillance methods, and the low surveillance rates, there is a significant clinical need for novel minimally invasive testing to aid in the detection of HCC at an early stage. Efforts to address this need include discovery and development of novel biomarkers [7], and diagnostic algorithms combining biomarker data and patient features to improve performance. As an example, patient sex and age combined with measurement of Lens culinaris agglutinin-reactive AFP (AFP-L3), AFP, and des-γ-carboxyprothrombin (DCP) was used to produce the diagnostic GALAD score. External validation for one model resulted in improved sensitivity of detection to the > 90% though specificity dropped to 62% [8]. When used in combination with ultrasound (GALADUS) detection is further enhanced, though this observation requires further external validation [9].

A recent innovation in the cancer diagnostics field has been the analysis of genetic or epigenetic cancer markers in patient plasma or serum. This approach, termed liquid biopsy, is becoming a standard clinical tool for the identification [10] and classification of cancer as well as an aid in treatment selection [11, 12]. This has been achieved by measuring circulating tumor DNA (ctDNA) [13, 14] in patient blood either by detection of genetic changes such as single nucleotide polymorphism (SNP) patterns or somatic gene mutations as in the recent example of the TERT C228T promoter mutation [15], or by identifying tumor associated DNA methylation patterns in cell free DNA (cfDNA) [16]. For example, previous studies showed that the presence of methylated SEPTIN9 (mSEPT9) DNA in plasma cfDNA was correlated with the occurrence of HCC in patients with cirrhosis [17, 18]. The SEPTIN9 gene encodes Septin-9, a member of the conserved septin family of GTP-binding proteins that function in key processes including vesicle trafficking, apoptosis, cytoskeletal remodeling and cell division [19]. The Septin-9 protein also acts as a tumor suppressor, regulating orderly and controlled cell growth.

The primary objective of this study was to determine the performance of mSEPT9 (commercially known as HCCBloodTest) and a novel methylated DNA biomarker panel for HCC surveillance. These assays were tested using plasma cfDNA from well characterized cirrhotic patients with and without early stage HCC Barcelona Clinic Liver Cancer (BCLC, stages 0, A, B) as the objective of HCC surveillance is the detection of patients with early stage disease. In addition, we report the results of a post-hoc analyses combining the NGS panel of gene methylation markers with AFP measurement for HCC surveillance in this patient population.

## Methods

### Design

The study was designed to determine the performance of mSEPT9 and a novel next generation sequencing (NGS) DNA methylation panel (without mSEPT9) as surveillance biomarkers for HCC detection in patients with cirrhosis. The biomarker panel was first established and the interpretive algorithm trained using plasma DNA samples from cirrhotic patients with and without cirrhosis. mSEPT9 was used in the HCCBloodTest format (Epigenomics AG Berlin Germany) following the instructions for use for the kit. The performance of the methylation panel and HCCBloodTest were then tested using plasma samples from a cross-sectional case control study of well characterized cirrhotic patients with no HCC or early stage HCC. Post hoc analyses were performed to assess the performance of the panel in combination with AFP measurement in this population.

### Patients

NGS Marker Discovery and Training: Sample collection protocols were approved by local Institutional Review Boards and all patients provided informed consent to participate in the studies. Plasma samples from 41 cirrhotic subjects with HCC and 46 subjects with cirrhosis who were negative for HCC were used for the training set (University Halle-Wittenberg, ethical approval No. 2012-5).

For testing, subjects were enrolled under a cross sectional case control study design registered on ClinicalTrials.gov (NCT03804593). Patients were enrolled at the University of Southern California (USC) Keck Medical Center and the California Liver Research Institute. The study collection protocol was approved by the Institutional Review Boards at both sites and patients provided written informed consent prior to study participation. Patients included men and women 18 years or older with Child class A or B scores. Exclusion criteria are provided in the Additional file 1. In addition to plasma sample collection, AFP measurements were also obtained for all subjects.

Controls (Group 1) included patients with a diagnosis of cirrhosis and no HCC as confirmed by 4 phase abdominal contrast-enhanced MRI or CT imaging performed ≤ 90 days prior to the date of consent or an abdominal contrast-enhanced MRI performed ≤ 45 days after enrollment. Patients had either no lesions or lesions with a liver imaging reporting and data system (LI-RADS) score of LR-1 or LR-2. All abdominal imaging was interpreted by one central radiologist. A total of 103 patients with Child class A or B met enrollment criteria for Group 1 and provided a sufficient plasma sample for the study. 102 of these were sufficient for additional research.

Cases (Group 2) included patients with a diagnosis of HCC that was confirmed by use of a 4-phase abdominal MRI or CT imaging performed ≤ 90 days prior to the date of consent or by use of a 4-phase abdominal MRI performed ≤ 45 days after enrollment with a LI-RADS category of LR-5 and/or biopsy with histopathology. All abdominal imaging was interpreted by one central radiologist. A total of 60 patients fulfilled the enrollment criteria and Barcelona Clinic Liver Cancer (BCLC) stage criteria (0/A/B) for Group 2 and provided a sufficient plasma sample.

### Sample preparation

Blood samples were drawn in EDTA plasma tubes, and plasma was prepared by centrifugation and further cleared by a second centrifugation. Plasma aliquots were stored at − 70 °C until processing.

Bisulfite treated DNA (bisDNA) was prepared from plasma with reagents from the commercially available Epi BiSKit (Epigenomics AG, Berlin) [20] using an automated protocol on the Tecan Evo 200 liquid handling platform (Männedorf, CH) [21]. In order to ensure that all samples from the same patient were comparable, multiple plasma samples from an individual patient were pooled and equally distributed into 3.5 ml aliquots, processed in parallel and stored at − 20 °C.

### Multiplex marker panel

The bisulfite targeted multiplex NGS panel as used for training, consisted of two methylation unspecific control DNA targets and 17 targeted methylation specific marker candidates (including SEPTIN9), ranging in amplicon size from 63 to 105 bases. The marker candidates used in the training panel originated from three different sources: 1. Six previously described cancer markers (including mSept9 and mRASSF2). 2. Five marker candidates originated from Epigenomics’ internal discovery by differential methylation hybridization (DMH) [22]. 3. Six candidates identified by in-silico discovery aiming for specific markers methylated in HCC but unmethylated in blood and preferably also unmethylated in other (solid) cancers, using public data from different sources. The final panel used in testing comprised the same markers, excluding SEPTIN9.

#### Multiplex PCR for NGS

The PCR was set up as one single reaction per sample using bisulfite DNA template from an equivalent of about 1 ml plasma in a ready to use multiplex PCR reaction (QIAGEN® Multiplex PCR) according to the manufacture’s recommended protocol. PCR primers were modified with a 5′phosphate for NGS library preparation. The multiplex PCR profile used a protocol as follows: denaturation at 94 °C for 30 s, annealing at 56 °C for 90 s, extension for 30 s at 72 °C; 45 cycles.

#### Library preparation and 2nd generation Sequencing

NGS library preparation was done according to the Illumina TruSeqNano DNA library preparation protocol [TruSeq®NanoDNALibraryPrep ReferenceGuide, Illumina] with the following modifications: The DNA fragmentation and end repair steps were skipped and DNA purification steps were adapted to isolate shorter DNA fragments. 15 µl of each multiplex PCR was purified using a magnetic beads/DNA ratio of 0.8 in the presence of 20% isopropanol. Purification steps after adenylation, adapter ligation and enrichment PCR were done with a magnetic beads/DNA ratio of 0.6 also in the presence of 20% isopropanol. The prepared libraries were quantified with the Qubit dsDNA HS Assay Kit (Thermo Fisher Scientific). Sequencing was done with MiSeq Reagent Kit v2 using a read length of 300 bp and targeting 200 k reads per sample.

#### NGS raw data interpretation

Paired Fastq files were trimmed to insertions between sequencing adaptors, and paired sequences were merged using flash [23]. All further data analysis was done in R [24] based on proprietary code using Rcpp [25] to increase processing speed and comprised the following steps: sequences were filtered for those flanked by primers on both sides reflecting molecules amplified by PCR, called Inserts. Inserts containing more cytosine than guanine outside of the CpG context were turned to their reverse complement to enable easy assessment of methylation by taking cytosine positions of CpGs into account exclusively. Such inserts were aligned to reference sequences of the assays to assess DNA-methylation: For each assay/sample combination any methylation pattern at CpG sites was assessed by counting occurrence of cytosines and thymidines at CpG positions. Comethylation within a single insert read was defined by cytosine in all or all except one CpG position (allowing an exception of one CpG to be different due to any error or SNP at a single CpG site) [26]. Quantitative co-methylation measured as the normalized number of co-methylated fragments of a marker in a sample was calculated as the number of comethylated insert sequences divided by the total number of all inserts found for a sample, normalized by the length of the sequences (in the following simply referred to as co-methylation).

#### Training the NGS panel

Training was performed by a simple, robust method applicable to small sample sets: marker candidate performance was characterized by receiver operating characteristic (ROC) differentiating groups of cirrhotic patients without HCC and those with HCC using quantitative co-methylation. N marker candidates with an area under the curve (AUC) of 0.7 were defined as usable markers. For each of the N markers a co-methylation cutoff was determined at a specificity of 0.9 that was used to determine whether a single marker was classified positive or negative. Marker panel measurements for a sample were defined as number of n positive markers. The marker panel characteristic was described by using n/N e.g. for ROC characteristics. The set of markers and their comethylation cutoffs from the training set were defined as the training result and stored in an R object.

### Assay performance

#### mSEPT9/HCCBloodTest

For the HCCBloodTest, bisDNA from each patient sample was analyzed as PCR triplicates on an ABI 7500 FAST Dx. The final test results of the HCCBloodTest were derived from the triplicate results by means of the algorithm defined in the respective Instructions for Use [27].

#### NGS panel

Bisulfite DNA from plasma samples were processed, measured and assessed blinded. For each sample each marker was binarized to be either positive or negative using the trained comethylation cutoffs leading to scores of n [0:7] positive markers for each sample. Patient group identity was then un-blinded and performance of the panel described as AUC and sensitivity/specificity at a cutoff of n+/7.

### Post-hoc analyses

Additional analyses were performed assessing a combination of mSEPT9 and AFP (20 ng/mL) as well as for the multi-marker NGS panel, including an alternative simple additive algorithm and a combination of the NGS results with AFP (20 ng/mL) to form a multiomic panel.

### Statistical analysis

Analysis was primarily descriptive and performed using standard libraries of the R environment [24]. If not stated otherwise, 95% confidence intervals are reported, and statistical tests were conducted at significance level 0.05.

## Results

Training samples included 46 cases and 41 controls. For testing, 61 patients with cirrhosis and HCC (cases) and 104 patients with cirrhosis and no detectable HCC (controls) were enrolled (Table 1). Excluded patients included one control patient with Child C cirrhosis; one patient with HCC who had a stage D (advanced) cancer by BCLC criteria; and for one control patient there was insufficient plasma for analysis using the additional NGS panel. Patient information including age, sex, Child class, etiology of cirrhosis and for HCC cases information on nodules and BCLC stage of cancer is summarized in Table 1. Additional patient information is provided in Additional file 1: Figure S1. There was a predominance of males among patients with HCC and they were older. The underlying etiologies of cirrhosis were non-alcoholic steatohepatitis (NASH), chronic viral infection (HBV, HCV) and alcoholic liver disease (ALD).

**Table 1:** Patient cohort characteristics of all enrolled patients including two that did not meet inclusion criteria for the final analysis

Variable	Patient without HCC (n = 104)	Patients with HCC (n = 61)	
Continuous	Median (min, max)	Median (min, max)	*p* value (test method)
Age (years)	57 (28–74)	64 (34–89)	< 0.001 (Wilcox)
Number of HCC nodules	–	1 (1–5)	–
Size of largest HCC nodule (mm)	–	27 (7–118)	–

Categorical	Count (%)	Count (%)	
Male sex	44 (42.3)	43 (70.5)	< 0.001 (Fisher)
Child-class			0.32 (Fisher)
A	60 (57.7)	41 (67.2)	
B	43 (41.3)	20 (32.8)	
C (excluded)	1 (1.0)		–
Etiology of cirrhosis			0.17 (Chi-square)
ALD	26 (25.0)	15 (24.6)	
HBV	2 (1.9)	4 (6.6)	
HCV	20 (19.2)	18 (29.5)	
NASH	33 (31.7)	17 (27.9)	
Other	23 (22.1)	7 (11.5)	
BCLC stage			
Stage 0	–	10 (16.4)	–
Stage A	–	39 (63.9)	–
Stage B	–	11 (18.0)	–
Stage D (excluded)		1 (1.6)	–

**Fig. 1:**
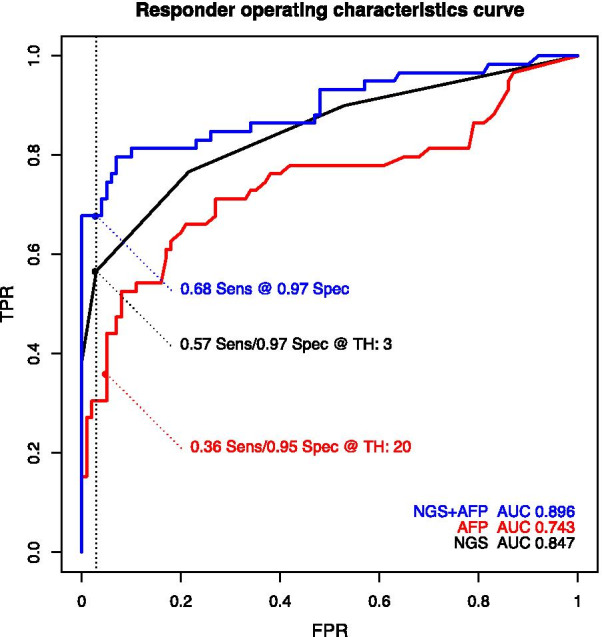
Receiver operating characteristic (ROC) analysis of the NGS Panel, AFP and AFP + NGS Panel. ROC curves for the NGS panel (black), AFP (red) and combination of NGS panel with AFP (blue) using logistic regression for 60 HCC versus 102 controls in the testing set. Areas under the curve (AUC) are written at the bottom right using the corresponding colors. Selected sensitivity/specificity pairs indicated on the curves are reported AFP ≥ 20 ng/mL, the NGS panel using the trained algorithm, and the post hoc combination of the NGS panel and AFP

### Panel training results

From the 16 marker candidates assessed in the targeted multiplex PCR panel, seven markers could be identified based on AUC ≥ 0.7: mASCL2, mLDHB, mLGALS3, mLOXL3, mPLXND1, mOSR1, mRASSF2 originated from different sources: RASSF2 is a previously known marker, mOSR1 is from Epigenomics’ discovery, the other five candidates were from in-silico discovery. All trained parameters were stored for use in the testing.

### Assay performance

#### HCCBloodTest: mSEPT9

Valid HCCBloodTest results were reported for all patient samples in the test set (Table 2). Among the 60 HCC cases, 46 tested positive, yielding a sensitivity of 76.7% (CI 95 64.6–85.6). Test results were also positive for 37 out of 103 cirrhotic patients without diagnosed HCC; observed specificity was 64.1% (CI 95 54.5–72.7).

**Table 2:** Summary of sensitivity and specificity of surveillance methods for early stage HCC detection in patients with cirrhosis

Surveillance assay	Sensitivity	Specificity
Test study		
HCCBloodTest	76.7% (CI 95 64.6–85.6)	64.1% (CI 95: 54.5–72.7)
NGS panel	57% (44–68)	97% (92–99)
AFP (20 ng)	36% (25–48)	95% (89–98)
Post hoc analyses		
AFP	31% (20–43)	97% (92–99)
mSEPT9 + AFP	42% (31–55)	97% (92–99)
NGS panel + AFP	68% (55–78)	97% (92–99)

A performance difference was observed among the various etiologies of chronic liver disease. For the 44 patients with chronic viral hepatitis-associated cirrhosis, the performance measures were 81.8% (18/22) sensitivity at 86.4% (19/22) specificity for HCC. For the 49 patients with NASH associated cirrhosis and HCC, sensitivity was observed at 87.5% (14/16), but specificity was only 39.4% (13/33).

#### NGS panel

For each patient sample a median of 204 k NGS reads were obtained and assessed. The overall performance, as described by receiver operating characteristic curves found for testing (see Fig. 1), was similar to that observed from training. The AUC was 0.85 (95% CI 0.78–0.91) and based on the trained algorithm we observed a sensitivity of 57% (95% CI 0.44–0.68) at 97% specificity (95% CI 0.92–0.99), see Table 2. More granular data on performance by stage, etiology of cirrhosis and Child class status are provided in the Additional file 1: Table S1.

### Post hoc analyses

As part of the cross-sectional study, patient data included AFP results. For comparative purposes AFP (at a cut off ≥ 20 ng) had a sensitivity of 36% at a specificity of 95%. Further exploratory analyses were performed to assess the potential of multiomic panels including the methylation markers and AFP. Using the NGS marker panel in combination with AFP (logistic regression) lead to AUC of 0.9 (95% CI 0.84–0.95) and sensitivity of 68% at 97% specificity.

## Discussion

In the current cross-sectional study, we report on the performance of DNA methylation biomarkers isolated from cell free plasma DNA for the surveillance of patients with liver cirrhosis to detect HCC at an early stage. Cases comprised a cohort of patients with liver cirrhosis and early-stage HCC based on BCLC classification, representing the target surveillance population. Similarly, controls in the study comprised cirrhotic patients of the same Child classes, for whom no detectable HCC was present based on ultrasound imaging that was confirmed by MRI or CT.

In this Southern California population of patients, we observed an overall sensitivity of 76.7% at a specificity of 64.1% for mSEPT9 using the HCCBloodTest. These results are comparable to those reported by Kotoh et al. [18] for a mSEPT9 assay with a sensitivity of 62.5% for HCC at a specificity of 71.7% among cirrhotic patients. However, their study also included BCLC Stage C patients and enrolled exclusively in Japan, with a greater proportion of patients having chronic viral infection compared to our study. Similar to Kotoh et al., we observed improved HCC detection with more advanced stages of HCC. Our test sensitivity was also comparable to that reported by Oussalah et al. for BCLC Stage A patients (72.73%), though the specificity reported was higher in their study (86.4%) [17]. While the underlying reasons for differences between the studies are not completely clear, and the sample size of the current study does not allow for detailed subgroup analysis, we did observe population differences. In the current study, there was a greater proportion of female subjects, fewer patients with BCLC stage B HCC and the spectrum of cirrhosis etiologies differed, with a greater proportion of NASH subjects in the non-HCC class. In this regard, we did observe substantial differences with respect to the specificity of the mSEPT9 assay, in particular lower performance in patients with NASH.

We also report on a panel of additional methylation biomarkers analyzed by bisulfite DNA NGS. These markers were initially selected and trained with an independent set of cases and controls, then tested using a fixed algorithm in a blinded fashion using the same patient collection as used for the HCCBloodTest. In this patient population the performance of the NGS panel showed improvement when compared with AFP. This outcome with epigenetic markers compares well with a recent report on hydroxymethylation where an AUC of 0.846 was observed for a 32-marker panel comparing patients with early stage HCC versus those with cirrhosis/chronic hepatitis [28]. Similarly, Hlady et al. [29] report good performance with a panel comprising hypo and hypermethylated CpG sites, though on a small sample size. Taken together, these reports suggest the potential for epigenetic markers measured in plasma cfDNA to aid in early detection of HCC in a surveillance program for patients with cirrhosis.

As part of a post-hoc analysis, we also combined the AFP outcomes with the methylated biomarker panel data to produce a multiomic panel which resulted in a sensitivity of 68% at a specificity of 97%. These outcomes compare favorably with other reported methods discussed above. While clearly exploratory in nature, these data provide direction for future efforts to further improve the assay, and assess the marker combinations in a new independent test set. These data support the potential for this DNA methylation panel as a simple marker panel for early stage HCC detection. Performance compares well with other liquid biopsy assays. Exploratory inclusion of additional parameters such as age and sex, as outlined for GALAD, using logistic regression lead to increased AUCs but might be biased by over fitting within this limited data set.

The study had a few limitations. The sample size was limited, precluding a detailed sub-group analysis. However, the objective of this study was an assessment of methylation markers for early stage detection, and as such, the aggregate sample size was sufficient for this assessment. In addition, measurement of AFP-L3 and DCP were not available, limiting comparison to AFP but not the GALAD model. Finally, the cross-sectional design allows immediate assessment of the biomarkers for HCC detection. However, false positives in this design may resolve into true positives over time, if the markers are associated with the earliest stages of HCC development, that are not detectable by imaging. Nonetheless, the current design identified markers with surveillance potential for future analysis.

## Conclusions

In this study, we demonstrated 57% detection of early stage HCC at an acceptable false positive rate of 3% for blood-based testing using methylation markers with a pretrained algorithm in an independent test set of well characterized cirrhotic patients with or without early stage HCC. This level of clinical performance can be achieved with a simple and affordable method and could be particularly applicable in settings where resources for surveillance by imaging may be limited. Furthermore, there is promise that the combination of the screening panel with other diagnostic parameters currently in use, such as AFP, may further enhance the performance. Though such findings require additional validation in an independent cohort, the results of this study support further development of cfDNA methylation markers for HCC surveillance in cirrhotic patients.

## Supplementary Information

**Additional file 1:** The supplement includes study design details, further patient characteristics, and additional performance data.

## Data Availability

The datasets used and/or analyzed during the current study are available from the corresponding author on reasonable request. Compiled data used for in silico discovery of the marker candidate panels assessed in this study comprised data sets that were obtained from the National Center for Biotechnology Information (NCBI) GEO database (https://www.ncbi.nlm.nih.gov/geo/) (Edgar R, Domrachev M, Lash AE. “Gene Expression Omnibus: NCBI gene expression and hybridization array data repository” Nucleic Acids Res. 2002 Jan 1;30(1):207–10), thereof relevant for this study: Shimada et al., 2019, accession IDs GSE112791 and GSE113019; Horvath et al*.* 2014, accession ID GSE61258; accession ID GSE75041; Lehne et al. 2015 and Wahl et al. 2017 accession ID GSE55763; and Zaimi et al. 2018 accession ID GSE123914.

## Abbreviations

HCC: Hepatocellular carcinoma
AFP: Alpha fetoprotein
NGS: Next generation sequencing
BCLC: Barcelona clinic liver cancer staging system
AUC: Area under the curve
ctDNA: Circulating tumor DNA
cfDNA: Cell free DNA
bisDNA: Bisulfite treated DNA
mSEPT9: Methylated SEPTIN9
PCR: Polymerase chain reaction
MRI: Magnetic resonance imaging
CT: Computed tomography
NASH: Non-alcoholic steatohepatitis
ALD: Alcoholic liver disease
CI: Confidence interval
