# Supplementary material for: Plasma cell free DNA methylation markers for hepatocellular carcinoma surveillance in patients with cirrhosis: a case control study
Source: BMC Gastroenterol. 2021 Mar 25;21:136. doi: 10.1186/s12876-021-01714-8 (PMC7995734; doi:10.1186/s12876-021-01714-8)
Supplement: Supplementary file 1 — Additional file 1: The supplement includes study design details, further patient characteristics, and additional performance data. [file 12876_2021_1714_MOESM1_ESM.docx]

**Supporting Information**:

Plasma cell free DNA methylation markers for Hepatocellular Carcinoma surveillance in patients with cirrhosis.

Patients – Training Set (3 from BioSophia, 38 from Halle)

Patients – Testing Set – Inclusion Criteria

Both Groups:

- Men or women age 18 years or older;
- Able to read, understand and sign informed consent to participate in study;
- Willing and able to provide written informed consent;
- Willing and able to meet all study requirements and undergo venipuncture to provide blood samples;
- Child class A or B.

Group 1:

Diagnosis of cirrhosis and no HCC confirmed by an abdominal contrast-enhanced MRI or CT imaging performed ≤ 90 days prior to the date of consent or an abdominal contrast-enhanced MRI performed ≤ 45 days after Visit 1. Patients with no lesions or lesions with an LI-RADS score of LR-1 or LR-2.

Group 2:

Diagnosis of HCC confirmed by an abdominal contrast-enhanced MRI or CT imaging performed ≤ 90 days prior to the date of consent or an abdominal contrast-enhanced MRI performed ≤ 45 days after Visit 1 with LI-RADS score of LR-5 and/or biopsy with histopathology.

Exclusion Criteria

Both Groups:

- Child class of C;
- Subject has undergone a colonoscopy, endoscopy or other invasive diagnostic procedure (other than venipuncture) during the 10 days prior to providing a blood sample for this study;
- Pregnancy;
- Breastfeeding;
- Currently undergoing dialysis;
- Currently receiving investigational treatments of any type;
- History of receiving any drug therapy, surgery or liver transplant for the treatment of HCC;
- Diagnosis of any non-HCC cancer (other than non-melanoma skin cancer) within past 5 years and/or currently undergoing treatment for any cancer;
- Any clinical condition, diagnosis, or social circumstance that, in the opinion of the Investigator, would be mean participation in the study would be contraindicated.

**Group 1:**

- Diagnosis of HCC or imaging data indicative of anything other than no lesion or lesions with an LR-1 or LR-2 classification.

**Group 2:**

- Imaging classification of LR-1, LR-2, LR-3 or LR-4.

**FIGURES**

|  | **Supplement Figure 1:** Patient distribution of Class/Stage, Sex and Child class by different source of cirrhosis. |
| --- | --- |
| **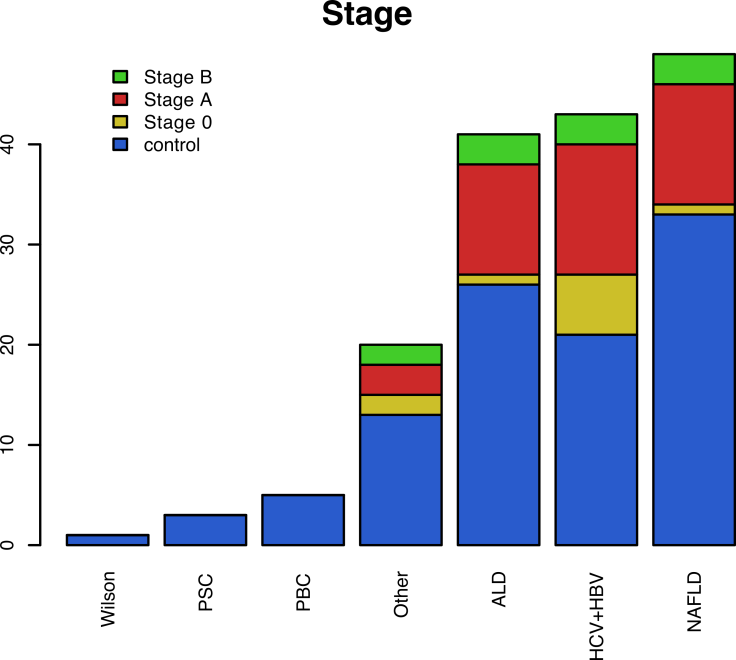**  **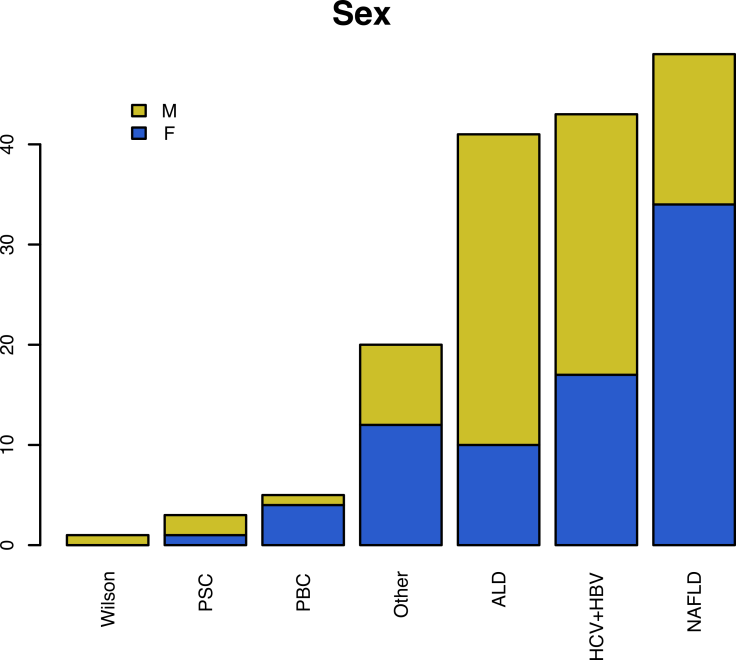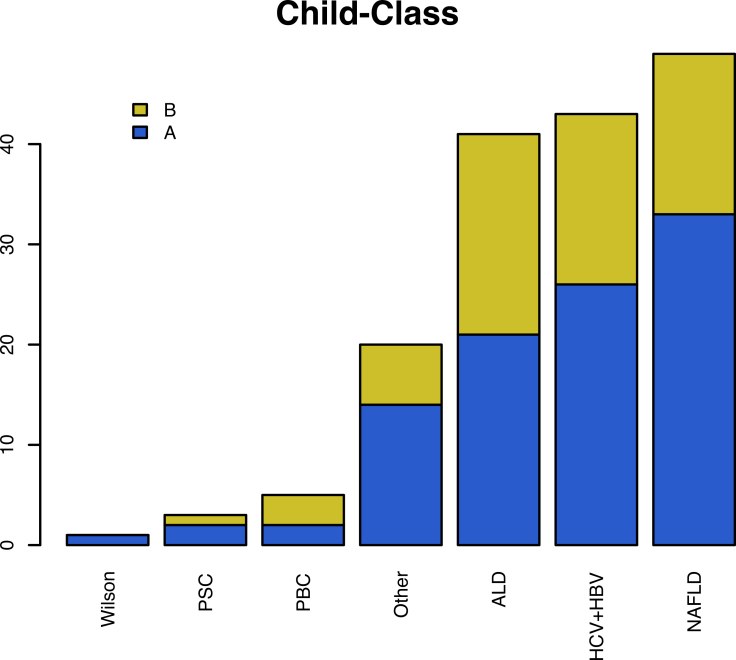** |  |

Supplement Table 1. Detailed performance of the NGS panel in comparison with AFP by Stage, Cirrhosis Etiology and Child-Pugh Score.
